# Influence of Surgical Technique on Mastectomy and Reexcision Rates in Breast-Conserving Therapy for Cancer

**DOI:** 10.1155/2012/725121

**Published:** 2012-01-16

**Authors:** Alison Unzeitig, Anne Kobbermann, Xian-Jin Xie, Jingsheng Yan, David Euhus, Yan Peng, Venetia Sarode, Amy Moldrem, A. Marilyn Leitch, Valerie Andrews, Roshni Rao

**Affiliations:** ^1^Division of Surgical Oncology, Department of Surgery, University of Texas Southwestern Medical Center, 5323 Harry Hines Boulevard, Dallas, TX 75390-9155, USA; ^2^Department of Clinical Sciences, University of Texas Southwestern Medical Center, 5323 Harry Hines Boulevard, Dallas, TX 75390-9155, USA; ^3^Department of Pathology, University of Texas Southwestern Medical Center, 5323 Harry Hines Boulevard, Dallas, TX 75390-9155, USA

## Abstract

*Introduction*. Breast conserving surgery (BCS) requires tumor excision with negative margins. Reexcision rates of 30–50% are reported. Ultrasound localization, intraoperative margin pathology, and specimen mammography have reduced reexcisions, but require new equipment. Cavity shave margin (CSM) is a technique, utilizing existing equipment, that potentially reduces reexcision. This study evaluates CSM reexcision impact. *Methods*. 522 cancers treated with BCS were reviewed. Patients underwent standard partial mastectomy (SPM) or CSM. Data collected included demographics, pathology, and treatments. *Results*. 455 SPMs were compared to 67 CSMs. Analysis revealed no differences in pathology, intraductal component, or neoadjuvant chemotherapy. Overall reexcision rate = 43%. Most reexcisions were performed for DCIS at margin. SPMs underwent 213 reexcisions (46.8%), versus 16/67 (23.9%) CSMs (*P* = 0.0003). Total mastectomy as definitive procedure was performed after more SPMs (*P* = 0.009). Multivariate analysis revealed CSM, % DCIS, tumor size, and race to influence reexcisions. *Conclusions*. CSM is a technique that reduces reexcisions and mastectomy rates.

## 1. Introduction

Nearly 200,000 women are diagnosed with breast cancer in the USA every year [1]. One accepted treatment for early-stage breast cancer is breast-conserving therapy (BCT), an option currently chosen by nearly half of all women [2]. For BCT to have equivalent survival to total mastectomy, all cancerous tissue must be removed with no evidence of tumor at the margins of resection, and adjuvant radiation therapy be given. Careful adherence to this oncologic approach also results in low rates of recurrence [3–5].

Although there remains controversy regarding what constitutes an acceptable microscopic margin of clearance, recent studies have revealed that the majority of surgeons prefer a 2 mm negative margin around the tumor [13]. With the advent of comprehensive pathologic analysis, careful scrutiny of all margins is routinely performed, and previous reports indicate that 30–50% of breast cancer patients undergo additional operations in order to obtain adequate margins [14–16]. Although these operations may be oncologically appropriate, it can be difficult for patients and does impose additional health care costs.

Techniques to facilitate complete removal of breast cancers with adequate margins at the initial operation following a diagnosis of breast cancer are of significant interest to surgeons and patients. Wire localization, a commonly used approach for localizing nonpalpable breast lesions, has been available since the early 1990s [17, 18]. It requires the placement of a thin wire into the lesion of concern, and although this does facilitate surgical excision, reexcisions are still commonly required [16, 18, 19]. Other techniques, including ultrasound guided hematoma localization [20, 21], seed localization [16, 19, 22, 23], radioguided localization [24–26], intraoperative specimen mammography [27, 28], and intraoperative pathologic margin assessment [29, 30] have also been utilized to ensure complete tumor removal. While success with these approaches has been reported, they universally require additional equipment and they may increase operative time.

A simpler technique that utilizes existing equipment available in any operative suite that may reduce the need for reexcision in breast conserving surgery (BCS) is performance of cavity shave margins (CSM). This technique has previously been described and is associated with decreased rates of reexcision [7, 9, 10, 12, 31, 32]. Surgeons either perform directed excisions of specific margins or excision of all margins adjacent to the lumpectomy cavity, not all patients in these reviews had a preoperative diagnosis of breast cancer, and some do not include the comprehensive pathologic analysis currently performed in the USA. This study evaluates the impact of routine CSM on reexcision rates in patients with a preoperative diagnosis of breast cancer utilizing current comprehensive microscopic pathologic examination.

## 2. Materials and Methods

An Institutional-review-board-approved retrospective analysis was performed to identify all patients undergoing BCT at an academic comprehensive cancer center. All patients had undergone core needle biopsy prior to surgery and had a known diagnosis of breast cancer; patients undergoing excisional biopsy for diagnosis alone were excluded. Patients underwent standard partial mastectomy with immediate CSM or PM with additional margins removed at the discretion of the surgeon (SPM). In patients who underwent CSM, after the initial partial mastectomy specimen was removed, Allis clamps were used to grasp the edges of the lumpectomy cavity, and six new margins (superior, inferior, anterior, posterior, medial, and lateral) were removed. These new margins were oriented with clips placed at the new margin and sent for permanent section; shave margins were at least 1 cm in thickness. If the initial lumpectomy specimen included pectoralis fascia, the area of breast parenchyma surrounding this exposed muscle was excised and sent as the posterior shave margin. Specimen mammography is routinely performed for patients undergoing radiologic localization prior to surgery. Data collected included demographics, pathology, adjuvant therapies, attending surgeon, number of surgical interventions, and final treatment outcome. Initial review revealed 522 patients who had undergone BCT. In the statistical analyses, Fisher's exact test and student's *t*-test were performed in Tables 1 and 2. And multivariate logistic regression models were built to explore the association between the outcome variable “need for 2nd operation to achieve adequate margins” and the predicting variables, as shown in Table 3. In the multivariate logistic regression, the stepwise model selection method was used with variables of *P* value less than 0.20 to enter the model and less than 0.05 to stay in the model. All reported *P* values are two sided. All statistical analyses were performed using SAS 9.2 for Windows (SAS Institute Inc., Cary, NC, USA).

## 3. Results

### 3.1. Rate of Reexcision

522 patients were included in the final analysis, 455 patients had undergone SPM, and 67 underwent CSM. Demographics, tumor pathology, age, and tumor size were equivalent between the two groups (Table 1). Specifically, factors such as lobular histology and presence of DCIS, which have previously been found to be associated with increased reexcision rates [33, 34], were not overrepresented in either group. Overall, 43.1% (*n* = 229) required a 2nd operation to obtain adequate margins. Patients who underwent CSM had significantly lower rates (23.9%  *n* = 16) of reexcision when compared to patients who underwent SPM (46.8%  *n* = 213) (Table 2).

### 3.2. Rationale for Reexcision and Residual Tumor Burden

**Figure 1:**
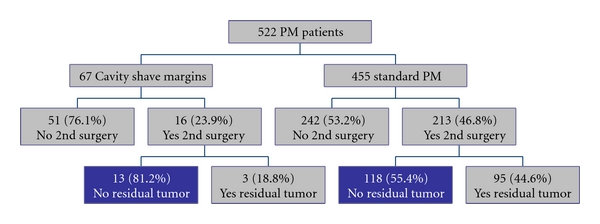
Reexcision rates of patients undergoing SPM versus CSM with analysis of residual tumor at reexcision.

Subsequently, an analysis of the rationale for reexcision and the risk of residual tumor burden was performed. For those patients who did require additional surgery after CSM, the majority (75%, *n* = 12) underwent reexcision for close margins (microscopically <2 mm margins), rather than positive margins which were more common (44.9%  *n* = 92) in the SPM group (Table 2). Of these reexcisions, a minority of patients in the CSM group (18.8%, *n* = 3) had residual tumor (Figure 1). In contrast, pathologic analysis of reexcisions in the SPM group revealed that 44.6% (*n* = 95) had residual tumor at the 2nd operation (Figure 1).

### 3.3. Mastectomy Rate and >2 Excisions

Despite initial plans for BCS, 78 (14.9%) of patients in this study eventually underwent total mastectomy for the treatment of their cancer. This change in therapeutic management was more common in the SPM group (16.5%, *n* = 75) versus the CSM patients (4.5%, *n* = 3). Data regarding the rationale for the change in surgical approach is not included in this study. Additionally, there were no patients in the CSM group that required >2 operations, in contrast to 10.1% (*n* = 46) of patients in the SPM who underwent >2 operations.

### 3.4. Multivariate Analysis

Upon multivariate analysis, CSM was the strongest controllable factor associated with complete removal of the primary tumor at the initial operation (Table 3). Additional factors contributing to lower reexcision rates included a lower percentage of DCIS, directed excision of additional margins based on surgeon discretion, smaller tumor size, and White race. Surgeons primarily removed additional directed margins based on evaluation of the specimen mammogram. Patients are given adjuvant chemotherapy based on National Comprehensive Cancer Network Guidelines.

## 4. Discussion

Previous studies evaluating the impact of multiple operations reveal dissatisfaction of the patient both physically and psychologically [32]. Physically, the patient may have an unsatisfying cosmetic outcome and is subjected to the increased length of recovery associated with additional surgery. Psychologically, the patient can lose confidence in the surgeon and fear recurrence. Ideally, a patient would go to surgery only once, achieving adequate margins and not returning to the operating room. Reexcision at a second operation potentially increases the likelihood of a poor cosmetic outcome and requires the patient to assume the risks of another surgical procedure under anesthesia.

CSM is a simple technique that utilizes existing equipment to remove extra margins of tissue after the primary breast specimen has been removed. Removal of six new margins (superior, inferior, anterior, posterior, medial, and lateral) provides an extra sampling of tissue that has been shown to reduce reexcision rates in patients undergoing BCS for breast cancer [7, 9, 12, 30–32]. Other studies have reported on groups of women who underwent CSM and compared lumpectomy margin status to shave margin status. These studies showed that overall final margin status was histologically negative in >50% of patients with histologically positive lumpectomy margins; therefore, a reexcision was avoided in these patients (Table 4) [6, 7, 9, 12]. The current study compared the reexcision rates before and after the introduction of routine CSM to primary BCS with additional margins taken at surgeon discretion. The reexcision rate fell significantly from 46.8% to 23.9% (22.9% reduction) after introduction of CSM. Other studies that also implemented CSM and compared reexcision rates to standard PM alone report similar reduction in reexcision rates from 7 to 30% (Table 4) [8, 10, 12]. Prior studies included patients who were undergoing excisional biopsy for diagnosis and varying approaches to what constituted an acceptable margin. In contrast, all patients in this series had a preoperative diagnosis of breast cancer via core needle biopsy, and patients were almost universally returned to the operating room for margins that were less than 2 mm. The significant reduction in reexcisions supports the use of CSM in the contemporary breast practice setting.

Furthermore, close (75% in CSM group compared to 44.9% in SPM group), rather than positive margins (25% in CSM group compared to 55.1% in SPM group) were the most common reason for a second operation in the CSM group. This could imply that CSM removes more cancerous tissue and thereby decreases the overall tumor burden left behind, a factor which may decrease the risk of recurrence upon long-term followup.

Another statistically significant difference was in the performance of a total mastectomy as definitive treatment; patients who underwent an SPM only were more likely to eventually choose mastectomy as a final operation (16.5% in SPM group compared to 4.5% in CSM group). This observed difference may be due to the amount of operations required to achieve adequate margins; more patients in the SPM group required >2 operations to achieve adequate margins, 10.1% in SPM group compared to 0% in CSM group. Several recent studies reveal increasing rates of prophylactic contralateral total mastectomy [35, 36]; the etiology of this trend continues to be unclear. In light of the current data, one of the factors that may be contributing to this increased mastectomy rate is the failure to successfully undergo BCS with one operation. As a cancer center policy, all eligible patients are offered BCT and reexcisions are routinely discussed and presented as an option to patients who fail to achieve adequate margins at the initial operation. The higher mastectomy rate in the SPM group may be a reflection of a loss of confidence in BCS as a therapeutic approach, and the patients desire to minimize the number of surgical interventions. Further investigation into the rationale for performance of the mastectomy in the SPM group as well as a potential association with contralateral prophylactic mastectomy is ongoing.

Patients with larger tumors required more reexcisions; this is possibly due to surgeons attempting to conserve more breast tissue at the initial operation, and having to go back for microscopic margins. Alternatively, this finding may be due to biological factors that lead to underestimation of larger tumors by imaging and clinical approaches, as well as growth patterns that favor occult tumor at the margins. This is also the likely explanation for the racial disparity, as AfricanAmerican and Hispanic patients typically present with larger tumors.

This study revealed that the majority of patients who required a reexcision in the CSM group and the SPM group did not have residual tumor. Although this is more evident in the CSM group (81.2% as opposed to 55.4% in the SPM group), it is still interesting to note that most of the patients in the SPM group may have been spared a second operation if CSMs were performed at the initial operation, as these patients did not have residual tumor. Given this finding, a cost analysis utilizing Current Procedural Terminology code 19301 was performed. If CSM was performed at the initial operation in those patients who underwent SPM, then 118 patients would have been spared a second operation, translating into a $183,018 surgical savings (2009, Medicare reimbursement). 

## 5. Conclusion

In conclusion, in the current era of preoperative core needle biopsy and comprehensive pathologic analysis, this study supports the use of CSM. It is an accessible and easily implemented surgical technique that, when compared to patients undergoing an SPM only, is associated with significantly reduced reexcision rates and decreased rates of total mastectomy. Further studies to evaluate the impact of this technique on recurrence rates, tissue volume removed, and cosmesis are ongoing.

## Figures and Tables

**Table 1:** Patient, tumor, and therapy factors.

	All patients *N* (%)	Cavity shave margins *N *(%)	Standard PM *N *(%)	*P* value
*Patient demographics*				
Race				
White	251 (48.1)	27 (40.3)	224 (49.2)	0.1913
Other	271 (51.9)	40 (59.7)	231 (50.8)	
Age at diagnosis	57	58	57	0.5835
BMI	29 kg/m^2^	30 kg/m^2^	29 kg/m^2^	0.107
Method tumor detected				
MMG/ultrasound, Combination, other	350 (67.0)	48 (71.6)	302 (66.4)	0.4865
BSE, PE	172 (33.0)	19 (28.4)	153 (33.6)	
*Tumor characteristics*				
Histology				
IDC	316 (60.5)	39 (58.2)	277 (60.9)	0.6896
Other	206 (39.5)	28 (41.8)	178 (39.1)	
Associated LVI				
Yes	52 (13.5)	5 (10.2)	47 (13.9)	0.6541
No	334 (86.5)	44 (89.8)	290 (86.1)	
ER status				
Positive	394 (76.4)	53 (79.1)	341 (75.9)	0.6456
Negative	122 (23.6)	14 (20.9)	108 (24.1)	
Her 2 neu status				
Positive	89 (17.8)	10 (15.4)	79 (18.1)	0.7282
Negative	412 (82.2)	55 (84.6)	357 (81.9)	
DCIS in final specimen				
Yes	384 (73.6)	47 (70.1)	337 (74.1)	0.5529
No	138 (26.4)	20 (29.9)	118 (25.9)	
% DCIS				
<25%	177 (46.1)	20 (42.6)	157 (46.6)	0.6419
>25%	207 (53.9)	27 (57.4)	180 (53.4)	
*Therapy type*				
Neoadjuvant chemo/hormonal Therapy				
Yes	57 (10.9)	9 (13.4)	48 (10.5)	0.5276
No	465 (89.1)	58 (86.6)	407 (89.5)	
Adjuvant chemo/hormonal Therapy				
Yes	205 (39.3)	29 (43.3)	176 (38.7)	0.504
No	317 (60.7)	38 (56.7)	279 (61.3)	
*Surgical localization*				
Breast surgery localization				
Wire	366 (70.8)	44 (66.7)	322 (71.4)	0.469
No localization	151 (29.2)	22 (33.3)	129 (28.6)	

PM: partial mastectomy; BMI: body mass index; MMG: mammogram; BSE: breast self exam; PE: physician exam; IDC: invasive ductal carcinoma; LVI: lymphovascular invasion; ER: estrogen receptor; Her 2 neu: human epidermal growth factor 2; DCIS: ductal carcinoma insitu; Chemo: chemotherapy.

**Table 2:** Statistically significant differences.

	All patients * N *(%)	Cavity shave margins* N *(%)	Standard PM * N *(%)	*P* value
Need for 2nd Operation to Achieve adequate margins?				
Yes	229 (43.9)	16 (23.9)	213 (46.8)	0.0003
No	293 (56.1)	51 (76.1)	242 (53.2)	
Reason for 2nd operation				
Positive margin	117 (52.9)	4 (25)	113 (55.1)	0.0346
Close margin (<2 mm)	104 (47.1)	12 (75)	92 (44.9)	
If close, type of tumor at margin				
DCIS	70 (65.4)	9 (75)	61 (64.2)	0.5377
Other	37 (34.6)	3 (25)	34 (35.8)	
If 2nd operation, was residual tumor present?				
Yes	98 (42.8)	3 (18.8)	95 (44.6)	0.0644
No	131 (57.2)	13 (81.2)	118 (55.4)	
Mastectomy eventually performed?				
Yes	78 (14.9)	3 (4.5)	75 (16.5)	0.0091
No	444 (85.1)	64 (95.5)	380 (83.5)	
>2 operations required to clear margins?				
Yes	46 (8.8)	0 (0)	46 (10.1)	0.0021
No	476 (91.2)	67 (100)	409 (89.9)	

PM: partial mastectomy; DCIS: ductal carcinoma insitu.

**Table 3:** Multivariate analysis.

	Odds ratio (95% CI)	*P* value
Race		
White	Reference	
Black	2.075 (1.129–3.815)	0.0125
Other	2.367 (1.220–4.589)	
Largest clinical diameter (cm)		
Continuous	1.288 (1.069–1.552)	0.0250
Shave margins taken		
Yes	0.229 (0.097–0.537)	0.0028
No	Reference	
Additional margins taken		
Yes	0.504 (0.292–0.871)	0.0054
No	Reference	
Percentage of DCIS		
<25%	Reference	<0.0001
>25%	4.655 (2.523–8.589)	

DCIS: ductal carcinoma insitu.

**Table 4:** Comparison of various studies.

	Preoperative diagnosis of breast cancer	*N* for CSM	*N* for standard PM	Number of CSM	Definition of negative margin	Reduction in reexcision	*P* value
Cao et al. [6]	Unknown	126	N/A	4–6	2 mm	61/103^a^	
Hewes et al. [7]	Yes	957	N/A	4	1 mm	107/196^b^	
Huston et al. [8]	Yes	45	49	4–6	2 mm	21%^c^	
Jacobson et al. [9]	Unknown	125	N/A	4–6	2 mm	61/83^d^	
Marudanayagam et al. [10]	Yes	394	392	4	Absence of tumor at resected margin	6.92%^e^	<0.01
Rizzo et al. [11]	Yes	121	199	4–5	1 mm	27.90%^f^	<0.05
Tengher-Barna et al. [12]	Yes	107	N/A	4	3 mm	24/47 ^g^	

CSM: cavity shave margin; PM: partial mastectomy.

^a,b,d,g^The overall final shave margin status was histologically negative in said amount of patients with histologically positive lumpectomy margins; therefore, ^a^reexcision was avoided in these patients.

^c^Reexcision rate in PM group 38.7% versus 17.7% in CSM group.

^e^Reexcision rate in PM group 12.5% versus 5.58% in CSM group.

^f^Reexcision rate in PM group 85.1% versus 57.2% in CSM group.
